# A Novel Aromatic Carboxylic Acid Inactivates Luciferase by Acylation of an Enzymatically Active Regulatory Lysine Residue

**DOI:** 10.1371/journal.pone.0075445

**Published:** 2013-09-16

**Authors:** Madoka Nakagomi, Nobuko Fujimaki, Ai Ito, Takahiro Toda, Hiroshi Fukasawa, Koichi Shudo, Ryoichi Tomita

**Affiliations:** 1 Department of Biology, Research Foundation Itsuu Laboratory, Tokyo, Japan; 2 Department of Chemistry, Research Foundation Itsuu Laboratory, Tokyo, Japan; 3 Molecular Function Department, Institute of Medicinal Molecular Design, Inc., Tokyo, Japan; University of East Anglia, United States of America

## Abstract

Firefly luciferase (Luc) is widely used as a reporter enzyme in cell-based assays for gene expression. A novel aromatic carboxylic acid, F-53, reported here for the first time, substantially inhibited the enzymatic activity of Luc in a Luc reporter screening. Matrix-assisted laser desorption/ionization time-of-flight mass spectrometry (MALDI-TOF-MS) and tandem mass spectrometry (MS/MS) analyses showed that F-53 modifies Luc at lysine-529 via amidation of the F-53 carboxyl group. The lysine-529 residue of Luc, which plays a regulatory catalytic role, can be acetylated. Luc also has a long-chain fatty acyl-CoA synthase activity. An *in vitro* assay that involved both recombinant Luc and mouse liver microsomes identified F-53-CoA as the reactive form produced from F-53. However, whereas the inhibitory effect of F-53 is observed in Hela cells that transiently expressed Luc, it is not observed in an *in vitro* assay that involves recombinant Luc alone. Therefore, insights into the activities of certain mammalian transferases can be translated to better understand the acylation by F-53. The insights from this study about the novel inhibitory modification mechanism might help not only to avoid misinterpretation of the results of Luc-based reporter screening assays but also to explain the pharmacological and toxicological effects of carboxylic acid-containing drugs.

## Introduction

Firefly luciferase (Luc) has been used in bioluminescence assays for high-throughput screening for small molecules with pharmacologically promising properties [1]–[3]. Luc is best known for its ability to catalyze the oxidation of firefly luciferin with molecular oxygen in the presence of adenosine triphosphate (ATP) and Mg^2+^, which results in the emission of yellow-green light [4]. However, Luc also possesses long-chain fatty acyl-CoA synthetic (ACSL) activity; it catalyzes the formation of fatty acyl-CoA via fatty acyl-adenylate from long-chain fatty acids in the presence of ATP, Mg^2+^, and CoA [5], [6].

Acyl-CoA synthetases (ACS) are encoded by 26 genes in the human genome; they catalyze the first step of fatty acid activation by forming a thioester with CoA, thus initiating pathways that synthesize structural lipids, signaling molecules, and other essential molecules [7]. There are five conserved amino acid sequence motifs in human ACS. The motif closest to the C terminus, motif V, is considered to have the potential to acetylate lysine [7]. In *Salmonella enterica*, ACS enzyme activity is posttranslationally regulated by acetylation of lysine-609, and activation of the acetylated enzyme requires the nicotinamide adenine dinucleotide (NAD^+^)-dependent protein deacetylase activity of the CobB Sir2 protein [8]. It has been suggested that acetylation modulates all members of the adenosine monophosphate (AMP)-forming family of enzymes, including Luc, which is acetylated at lysine-529 [8]. Indeed, virtually every enzyme that participates in glycolysis, gluconeogenesis, the tricarboxylic acid cycle, the urea cycle, fatty acid metabolism, and glycogen metabolism is acetylated in human liver tissue [9], [10]. However, the details of the acetylation mechanism(s) involved are not known [10].

A recent review emphasized that the interaction of small molecules with Luc can interfere substantially with the results of high-throughput screens that involve the Luc reporter [11]. For instance, although PTC124 inhibits Luc, it paradoxically increases cellular Luc activity in Luc reporter-based *in vitro* assays by increasing the posttranslational stabilization of Luc [12]. Therefore, Luc inhibition and stabilization result from the formation of an inhibitory product during the Luc-catalyzed reaction between ATP and PTC124 [12], [13]. However, we suggest that PTC124 affects not only Luc but also enzymes in mammals in the same manner as Luc; this is consistent with the fact that PTC124 has been developed as a suppressor of nonsense mutations that cause genetic diseases such as cystic fibrosis [14], [15]. We also identified novel synthetic small molecules that affected the enzymatic activity of Luc in a Luc reporter assay for the ligand screening of human retinoic acid receptor-related orphan receptor α1 (hRORα1) and were developed using the structural design of thalidomide derivatives as a reference [16]–[20]. Thalidomide is a teratogen for which the mechanism has long eluded identification because it binds to only one protein, Cereblon, which forms an E3 ubiquitin ligase complex [21]. However, thalidomide has been used in Japan since 2008 to treat patients with recurrent and intractable multiple myeloma. Meanwhile, it is suspected that carboxylic acid-containing drugs are able to become conjugated to biomolecules [22]–[24], although the actual site of creation of the adduct remains unclear. Therefore, we investigated the inhibition mechanism by which the aromatic carboxylic acid F-53 inhibits Luc. Our data reveal that unlike PTC124, F-53 inhibits Luc by covalent binding to a regulatory lysine residue of Luc in living mammalian cells.

## Materials and Methods

### Ethics statement

This study was carried out in strict accordance with the recommendations in the Guide for the Care and Use of Laboratory Animals of the National Institutes of Health. Animal protocols were approved by the Institutional Review Board of the Research Foundation Itsuu Laboratory (Permit Number: 11-3).

### Chemical synthesis

**Figure 1:**
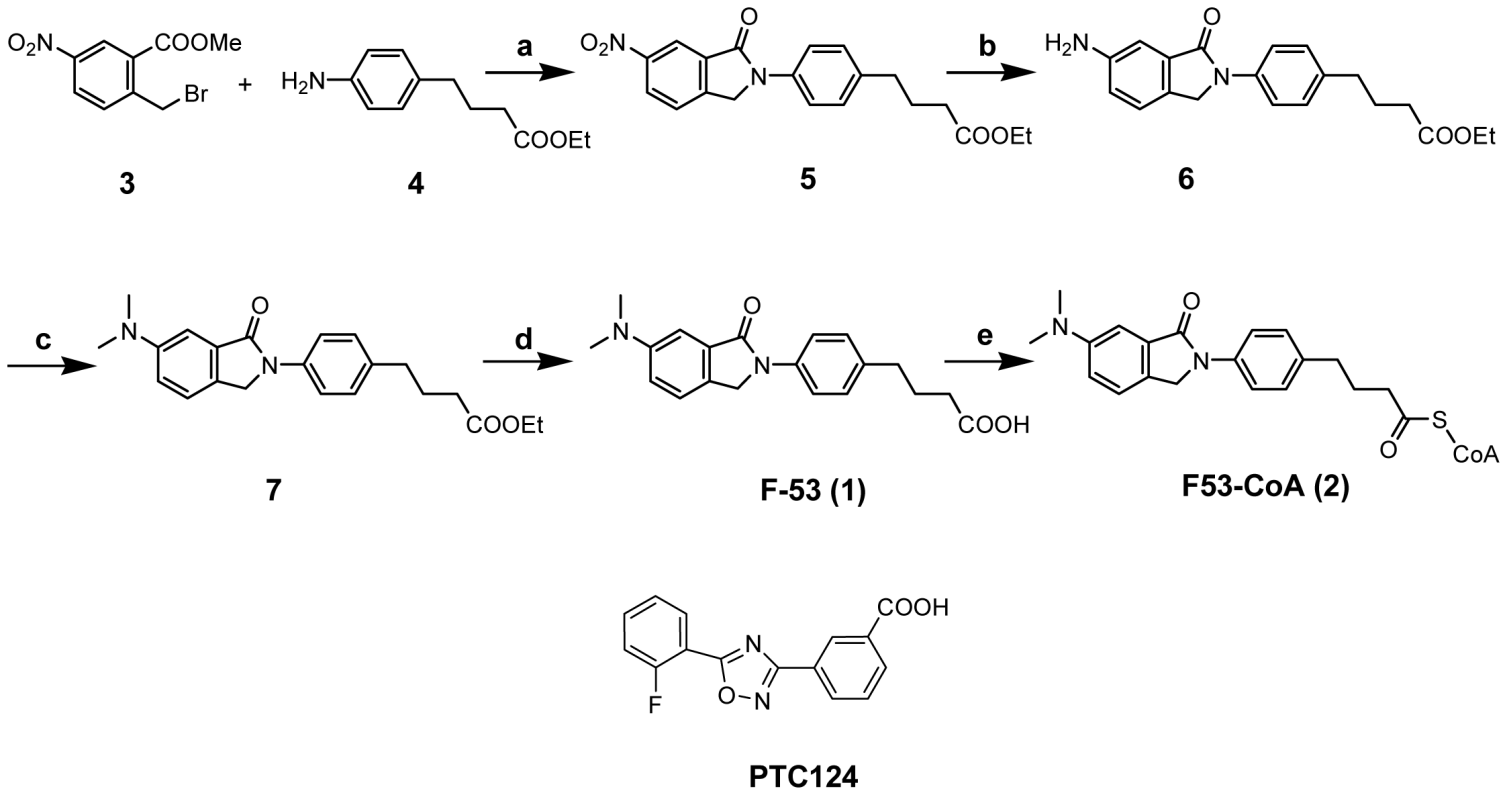
Chemical synthesis of F-53 (1) and its CoA conjugate (2), and the structure of PTC124. Conditions: a) pyridine, EtOH, reflux, 24 h, 62.3%; b) H_2_, 10% Pd-C, benzene, RT, 93.6%; c) HCHOaq., NaBH_3_CN, AcOH, THF, RT, 7.5 h, 88.4%; d) NaOH aq., EtOH, RT, 10 h, 77.6%; e) 3LiCoASH, PyBOP, K_2_CO_3_, THF, RT, 50%

The approaches used for the synthesis of F-53 (1) and its CoA-form (2) are shown in Figure 1.

#### General

All the solvents and chemicals used were reagent-grade commercial products. Analytical thin-layer chromatography was performed using Merck silica gel 60 PF_254_ plates (Merck Co., Tokyo, Japan). The plates were visualized by irradiation under a 254-nm UV lamp. Fuji Davison BW 200 (Fuji Silysia Chemical Ltd., Aichi, Japan) and silica gel was used for column chromatography. ^1^H-NMR spectra were recorded using a Varian Mercury 400 MHz spectrometer (Varian Medical Systems, Inc., Palo Alto, CA, USA). Chemical shifts are reported in ppm relative to the resonance of the solvent as a standard. Data are reported as follows: chemical shift, number of protons, multiplicity (s = singlet, d = doublet, dd = doublet of doublet, t = triplet, q = quartet, quint = quintuplet, br = broad, m = multiplet), and coupling constants. Melting points were determined using a Yanagimoto micro-melting point apparatus (hot plate; Yanaco New Science Inc., Kyoto, Japan) without correction. Mass spectra were recorded using a Hitachi M-80B spectrometer, with an ionizing voltage of 70 eV (Hitachi High-Technologies Co., Tokyo, Japan), with the numbers in parentheses indicating relative intensities. Infrared spectra were measured using a Shimadzu IR-460 spectrophotometer (Shimadzu Co., Kyoto, Japan).

#### 4-[4-(6-Nitro-1-oxo-1,3-dihydro-isoindol-2-yl)-phenyl]-butyric acid ethyl ester (5)

Pyridine (57 mg, 0.73 mmol) was added to a solution of 2-bromomethyl-5-nitrobenzoic acid methyl ester (0.15 g, 0.54 mmol) and 4-(4-aminophenyl) butyric acid ethyl ester (0.19 g, 0.90 mmol) in ethanol (EtOH, 10 mL), and the mixture was then stirred under reflux for 24 h. After cooling, the solution was poured into ice-cold water; the precipitated crystal was collected by filtration, washed sequentially with EtOH and then with water, and then dried. Recrystallization from acetone yielded **5** (0.12 g, 62.3%) as colorless needles or leaflets. MS (*m*/*z*): 368 (M^+^, 53), 280 (100); ^1^H-NMR (CDCl_3_) δ: 1.27 (t, 3H, *J* = 7.2 Hz), 2.00 (quint, 2H, *J* = 7.5 Hz), 2.34 (t, 2H, *J* = 7.5 Hz), 2.71 (t, 2H, *J* = 7.5 Hz), 4.16 (q, 2H, *J* = 7.5 Hz), 4.97 (s, 2H), 7.28 (d, 2H, *J* = 8.7 Hz), 7.71 (d, 1H, *J* = 8.4 Hz), 7.75 (d, 2H, *J* = 8.7 Hz), 8.48 (dd, 1H, *J* = 8.4 and 2.1 Hz), 8.76 (d, 1H, *J* = 2.1 Hz).

#### 4-[4-(6-Amino-1-oxo-1,3-dihydro-isoindol-2-yl)-phenyl]-butyric acid ethyl ester (6)

Compound **5** (0.12 g) was dissolved in benzene (30 mL) and was then catalytically reduced following the addition of 10% palladium on activated carbon (30 mg). The reaction mixture was filtered and evaporated under reduced pressure. The residue was recrystallized from EtOH to give **6** (0.11 g, 93.6%), a yellow-brown powder. MS (*m*/*z*): 338 (M^+^, 92), 237 (100); ^1^H-NMR (CDCl_3_) δ: 1.26 (t, 3H, *J* = 7.2 Hz), 1.96 (quint, 2H, *J* = 7.5 Hz), 2.33 (t, 2H, *J* = 7.5 Hz), 2.66 (t, 2H, *J* = 7.5 Hz), 3.87 (br, 2H), 4.13 (q, 2H, *J* = 7.5 Hz), 4.74 (s, 2H), 6.90 (dd, 1H, *J* = 8.4 and 2.1 Hz), 7.17 (d, 1H, *J* = 2.1 Hz), 7.22 (d, 1H, *J* = 8.1 Hz), 7.26 (d, 2H, *J* = 9.0 Hz), 7.75 (d, 2H, *J* = 9.0 Hz), 8.32 (d, 1H, *J* = 7.8 Hz), 8.46 (s, 2H), 8.54 (d, 1H, *J* = 7.8 Hz).

#### 4-[4-(6-Dimethylamino-1-oxo-1,3-dihydro-isoindol-2-yl)-phenyl]-butyric acid ethyl ester (7)

First, 95% sodium cyanoborohydride (0.13 g, 4.73 mmol) and acetic acid (0.1 mL) were added to a solution of **6** (0.11 g) dissolved in a mixture of formalin (0.37 mL) and tetrahydrofuran (THF) (5 mL). The mixture was stirred at room temperature (25°C±2°C) for 30 min before adding acetic acid (0.1 mL) and continuing to stir the mixture at room temperature for an additional 7 h. The reaction mixture was extracted twice with ethyl acetate (AcOEt). The organic layer was washed sequentially, first with 1 N potassium hydroxide (KOH) and then with a saturated sodium chloride (NaCl) solution, before drying over anhydrous sodium sulfate (Na_2_SO_4_). Evaporation of the solvent yielded a residue, which was purified using column chromatography with silica gel as the solid phase and hexane/AcOEt (2∶1, V/V) as the mobile phase, and was recrystallized from chloroform (CHCl_3_)/hexane to give **7** (0.10 g, 88.4%), a colorless powder. MS (*m*/*z*): 388 (M^+^, 100); ^1^H-NMR (CDCl_3_) δ: 1.26 (t, 3H, *J* = 7.2 Hz), 1.96 (quint, 2H, *J* = 7.5 Hz), 2.33 (t, 2H, *J* = 7.5 Hz), 2.66 (t, 2H, *J* = 7.5 Hz), 3.03 (s, 6H), 4.13 (q, 2H, *J* = 7.5 Hz), 4.75 (s, 2H), 6.96 (dd, 1H, *J* = 8.4 and 2.4 Hz), 7.21 (d, 1H, *J* = 2.4 Hz), 7.23 (d, 2H, *J* = 8.4 Hz), 7.34 (d, 1H, *J* = 8.4 Hz), 7.77 (d, 2H, *J* = 8.4 Hz).

#### 4-[4-(6-Dimethylamino-1-oxo-1,3-dihydro-isoindol-2-yl)-phenyl]-butyric acid (F-53, 1)

First, 0.5 M sodium hydroxide aq (1.9 mL) was added to a solution of **7** (70.5 mg, 0.19 mmol) in EtOH (15 mL); the mixture was then stirred at room temperature for 10 h. The reaction mixture was acidified with 10% hydrogen chloride, and then extracted twice with CHCl_3_. The organic layer was washed with a saturated NaCl solution, dried over anhydrous Na_2_SO_4_, and filtered. Evaporation of the solvent yielded a residue, which was in turn purified using column chromatography with silica gel as the solid phase and CHCl_3_/methanol (MeOH) (20∶1, V/V) as the mobile phase, and was recrystallized from EtOH to give **F-53** (50.5 mg, 77.6%), a pale brown powder. M.p. 186–187°C; MS (*m*/*z*): 338 (M^+^, 96), 55 (100); IR (KBr)cm^−1^ 3425, 1722, 1650, 1506, 1378, 1163, 808; ^1^H-NMR (CDCl_3_) δ: 1.99 (quint, 2H, *J* = 7.5 Hz), 2.39 (t, 2H, *J* = 7.5 Hz), 2.69 (t, 2H, *J* = 7.5 Hz), 3.03 (s, 6H), 4.75 (s, 2H), 6.97 (dd, 1H, *J* = 8.4 and 2.4 Hz), 7.21 (d, 1H, *J* = 2.4 Hz), 7.24 (d, 2H, *J* = 8.4 Hz), 7.34 (d, 1H, *J* = 8.4 Hz), 7.77 (d, 2H, *J* = 8.4 Hz).

#### F-53-CoA (2)

F-53 CoA was synthesized using a modification of a previously described method [25]. CoA trilithium salt (0.03 mmol, 23.6 mg), benzotriazole-1-yloxytrispyrrolidinophosphonium hexafluorophosphate (25.5 mg, 0.048 mmol), potassium carbonate (12 mg, 0.12 mmol), and **F-53** (15.2 mg, 0.045 mmol) were dissolved in 3 mL 50% THF/50% water and stirred at room temperature for 7 h. The reaction mixture was evaporated under reduced pressure to remove the THF organic solvent. The residual solution was then separated by using high performance liquid chromatography (HPLC)/ultraviolet (UV) (Waters Alliance e2695 separations module equipped with a 2489 UV detector; Nihon Waters K.K., Tokyo, Japan) along with a preparative reverse-phase HPLC column (Inertsil ODS-3 column, 4.6×250 mm, GL sciences Inc., Tokyo, Japan). A gradient of 10%–90% acetonitrile (CH_3_CN) in water, containing 10 mM ammonium acetate (NH_4_OAc), was used with a flow rate of 1 mL/min; elution was monitored at 260 nm. **F-53-CoA** was eluted with a retention time of 8 min. The fractions were combined and lyophilized. The final yield was approximately 59%. Mass analysis was performed using HPLC/mass spectrometry (MS) with positive-mode electrospray ionization (ESI) (Waters Alliance e2695 separations module equipped with a 3100 mass detector, Nihon Waters K.K. and a Cadeza CD-C18 HPLC column (2×150 mm, Imtakt Co., Kyoto, Japan) at 25°C. The mobile phase was 10%–90% CH_3_CN in water containing 10 mM NH_4_OAc (pH 7.0, flow rate 0.2 mL/min). MS (*m*/*z*): 1088 (M+1); ^1^H-NMR (CD_3_OD) δ: 0.82 (3H, s, Me), 1.07 (3H, s, Me), 1.95 (2H, quint, *J* = 7.2 Hz), 2.40 (2H, t, *J* = 6.6 Hz), 2.58 (2H, t, *J* = 7.2 Hz), 2.64 (2H, t, *J* = 7.2 Hz), 2.98 (2H, t, *J* = 6.6 Hz), 3.00 (6H, s, NMe×2), 3.18–3.36 (2H, m, overlapping with the solvent peak), 3.45 (2H, t, *J* = 6.6 Hz), 3.50–3.60 (1H, m), 3.98–4.10 (1H, m), 4.08 (1H, s), 4.24–4.32 (2H, m), 4.44–4.52 (1H, m), 4.83 (2H, s), 4.76–4.96 (2H, m), 6.11 (1H, d, *J* = 6.0 Hz), 7.09 (1H, dd, *J* = 8.4, 2.1 Hz), 7.12 (1H, d, *J* = 2.1 Hz), 7.25 (2H, d, *J* = 8.7 Hz), 7.43 (1H, d, *J* = 8.4 Hz), 7.74 (2H, d, *J* = 8.7 Hz), 8.20 (1H, s), 8.60 (1H, s).

#### GLTGK(F-53)LDAR

A suspension of *tert*-butoxycarbonyl (Boc)-GLT(*tert*-Butyl)GKLD(O-Butyl-*tert*)AR-2,2,4,6,7-pentamethyldihydrobenzofuran-5-sulfonyl (Pbf)-resin (78 mg, ca. 18 µmol, Sigma-Aldrich Japan Inc., Tokyo, Japan) in CH_2_Cl_2_ (2 mL) and N,N-dimethylformamide (1 mL) was cooled and added to **F-53** (31 mg, 92 µmol), 1-(3-dimethylaminopropyl)-3-ethyl-carbodiimide hydrochloride (18 mg), and 4-dimethyl-aminopyridine (1 mg). The mixture was gently stirred at room temperature for 1 d and filtered through a sintered-glass funnel. The residue was rinsed with CH_2_Cl_2_, THF, MeOH, and CH_2_Cl_2_ (82 mg). Deprotection solution (0.5 mL, 5% H_2_O, 25 mg dithiothreitol [DTT], 10 µL triisopropylsilane in trifluoroacetic acid [TFA]) was added and the mixture gently stirred at room temperature for 5 h, before being filtered through a sintered-glass funnel. The residual resin was rinsed with TFA (3 mL). The filtrate and rinsing solution were combined and concentrated to 0.3 mL under nitrogen gas. The solution was shaken after addition of ether (3 mL) and storage at −16°C for 30 min, before being centrifuged (800×*g*, 4°C, 10 min); the ether was then removed by pipetting. The precipitate was washed twice in the same manner: the sample was shaken after addition of ether (3 mL) and stored at −16°C for 30 min; this was followed by centrifugation (800×*g*, 4°C, 10 min) and removal of the ether by pipetting. The precipitate was dried to yield **GLTGK(F-53)LDAR**, a colorless powder.

#### PTC124

PTC124 was synthesized as described previously [12]–[14] and the structure is shown in Figure 1.

1H-NMR (*d6*-dimethyl sulfoxide [DMSO]) δ: 7.50 (dd, 1H, *J* = 7.8 and 7.8 Hz), 7.56 (dd, 1H, *J* = 10.8 and 7.8 Hz), 7.75 (dd, 1H, *J* = 7.8 and 7.8 Hz), 7.76–7.86 (m, 1H), 8.17 (ddd, 1H, *J* = 7.8, 1.5 and 1.5 Hz), 8.26 (ddd, 1H, *J* = 7.8, 7.8 and 1.2 Hz), 8.33 (ddd, 1H, *J* = 7.8, 1.5 and 1.5 Hz), 8.64 (dd, 1H, *J* = 1.5 and 1.5 Hz), 13.3 (br, 1H).

### Plasmids

Mammalian expression vectors for full-length hROR α1 (pcDNA3-hRORα1) and the thymidine kinase promoter-based Luc reporter plasmid (Rev-DR2×3-TK-Luc, which included three DR2 response elements) were generated using standard PCR-based cloning strategies. Mammalian expression vectors for full-length human retinoic acid receptor α (RARα; pSG5-hRARα) and the TK promoter-based chloramphenicol acetyl transferase (CAT) reporter plasmid (DR5G-TK-CAT, which included DR5) were kindly provided by Dr. Kagechika (Tokyo Medical and Dental University). The Luc+ expression plasmid pCMVluc+ was provided by the RIKEN BioResource Center (Ibaraki, Japan). pCMV-Tag 1, which produces N-terminally FLAG-tagged Luc protein, was purchased from Stratagene (La Jolla, CA, USA). The Renilla luciferase (RL) expression plasmid, pRL-CMV, was purchased from Promega Co. (Madison, WI, USA). The β-galactosidase (β-gal) expression plasmid pCMVβ was purchased from Clontech (Palo Alto, CA, USA). pUC19 was obtained from Bayou Biolabs (Metairie, LA, USA). The K529A, K529E, and K529R Luc mutations in the pCMV-Tag 1 control vector were generated using the QuikChange® II XL Site-Directed Mutagenesis Kit (Stratagene).

### Cell culture

COS-1 and HEK293 cells were provided by Institute of Medicinal Molecular Design, Inc. (Tokyo, Japan) and were cultured in Dulbecco's modified Eagle medium (DMEM) supplemented with 4.5 g/L D-glucose (DMEM high glucose; GIBCO, Life Technologies Japan Ltd, Tokyo, Japan) and 10% heat-inactivated fetal bovine serum (FBS; GIBCO, Life Technologies Japan Ltd), and HeLa cells were provided by Institute of Medicinal Molecular Design, Inc. and were cultured in DMEM low glucose (GIBCO, Life Technologies Japan Ltd) with 10% FBS, in a humidified incubator at 37°C and 5% CO_2_.

### Reporter assays

In the hRORα1 assay with the Luc reporter, HeLa cells were seeded at a density of 1×10^4^ cells/well in 96-well plates and transfected a day later with 20 ng/well pcDNA3-RORα1, 50 ng/well Rev-DR2×3-TK-Luc, 80 ng/well pUC19, and 10 ng/well pCMVβ to normalize differences in transfection efficiency. Simultaneous transfection with all four plasmids was performed using the FuGENE 6 Transfection Reagent (Roche Diagnostics K.K., Tokyo, Japan) in Opti-MEM® I Reduced Serum Medium (GIBCO, Life Technologies Japan Ltd). After 24 h, the transfected cells were treated with F-53 (10^−3^–10^−8^ M) for an additional 20–24 h before analysis. After removing the medium, the cells were treated with passive lysis buffer (PLB; Promega Co., Madison, WI, USA) for 30 min, and Luc activity was determined by measuring chemiluminescence after addition of PicaGene LT 2.0 (TOYO INK Co. Ltd., Tokyo, Japan). β-gal activity was determined by measuring fluorescence (465 nm) with a GENios instrument (Tecan Japan Co. Ltd., Kanagawa, Japan) after the addition of 4-methylumbeliferyl-β-D-galactopyranoside (MUG, Sigma-Aldrich Japan Inc, Tokyo, Japan). Protein concentrations were determined with a Micro BCA™ Protein Assay Kit (Thermo Fisher Scientific Inc., Rockford, IL, USA). For the CAT reporter RARα assay, COS-1 cells (5×10^5^ cells/well in 24-well plates) were simultaneously transfected with 30 ng/well pSG5-hRARα, 300 ng/well DR5G-TK-CAT, 120 ng/well pUC19, and 30 ng/well pCMVβ to normalize transfection efficiency. Transfections were performed using the FuGENE 6 Transfection Reagent in Opti-MEM® I Reduced Serum Medium. After 24 h, cells were treated with F-53 (10^−6^ M) or LE540 (10^−5^ M), with or without Am80 (10^−7^ M), for an additional 20–24 h. Subsequently, after the cells were rinsed three times with cold PBS (GIBCO, Life Technologies Japan Ltd), CAT activity was determined by monitoring absorption at 405 nm by using a CAT ELISA (Roche Diagnostics K.K.). β-gal activity and protein concentrations were detected as described above for hRORα1. For direct detection of changes in the cellular activities of Luc and other reporter enzymes, HeLa or HEK293T cells were transfected with pCMVluc+, pRL-CMV, the pCMV-tag1 control vector, or modified variants carrying Luc mutations. Cells were also transfected with pUC19 and pCMVβ to normalize for differences in transfection efficiency. All transfections were performed by using FuGENE 6 or the FuGENE HD Transfection Reagent (Roche Diagnostics K.K.) in Opti-MEM® I Reduced Serum Medium in 96-well plates. The cells were treated with F-53, PTC124, and various kinase inhibitors (Calbiochem, San Diego, CA, USA) at appropriate doses, at varying time points, and after various incubation periods. After removing the medium, the cells were treated with PLB for 30 min and Luc activity was determined by measuring chemiluminescence with a GENios instrument after addition of PicaGene LT 2.0. β-gal activity and protein concentrations were detected as described above for hRORα1.

### Reverse transcription-polymerase chain reaction (RT-PCR)

Total RNA was isolated from HeLa cells transiently transfected with pCMVluc+ by using the RNeasy® mini kit and RNase-Free DNase Set (QIAGEN K.K., Tokyo, Japan); 1 µg total RNA was reverse transcribed to cDNA by using the SuperScript III First-Strand Synthesis System for RT-PCR (Invitrogen, Life Technologies Japan Ltd). The cDNA template was PCR-amplified with specific oligonucleotide primers: *Luc*-For (5′-TCA AAG AGG CGA ACT GTG TG-3′), *Luc*-Rev (5′-TTT TCC GTC ATC GTC TTT CC-3′), human glyceraldehyde 3-phosphate dehydrogenase (*hGAPDH*)-For (5′-GAG TCC ACT GGC GTC TTC A-3′), and *hGAPDH*-Rev (5′-GGG CCA TCC ACA GTC TTC T-3′). PCR was performed using a Thermal Cycler PxE (Thermo Electron Co., Kanagawa, Japan) with a primer concentration of 10 µM in deionized distilled water (DDW). Each reaction contained cDNA derived from 50 ng RNA for *Luc* and 0.25 ng RNA for *hGAPDH*. PCR products were separated via gel electrophoresis in 2% agarose gels and visualized after staining with ethidium bromide. The gel image was exposed to Polaroid film (FP-3000B, Fujifilm, Tokyo, Japan). The gray value of each specific band was measured using an image analysis program (Scion Image, Scion Corporation). *Luc* expression was normalized relative to endogenous *hGAPDH* expression.

### Sodium dodecyl sulfate (SDS)- and native polyacrylamide gel electrophoresis (PAGE) and western blotting

HeLa cells were seeded at a density of 3×10^5^ cells/well in 6-well plates. After 24 h, cells were transfected with pCMVluc+, pCMVβ, and pUC19 by using FuGENE6. After another 24 h, the cells were treated with F-53, PTC124, or DMSO for 20–24 h, or were treated with F-53 or DMSO for 3.5 h with or without PD98059. The treated cells were washed twice with PBS, followed by addition of 10 mM tris(hydroxymethyl)aminomethane (Tris)-HCl (pH 7.5) containing 1% NP-40, 0.15 M NaCl, 1 mM ethylenediaminetetraacetic acid (EDTA), and protease inhibitors; the cells were then scraped into microtubes and resuspended by pipetting. The microtubes were kept on ice for 30 min and centrifuged at 17,000×*g* for 10 min at 4°C. After protein concentrations were determined with a Micro BCA™ Protein Assay Kit, the supernatants (12.5 µg protein/lane) were subjected to 7.5% native or SDS-PAGE. The recombinant Luc protein samples (0.2 ng) used in the inhibition assay *in vitro* were combined with the protein samples generated for native PAGE (12.5 µg protein/lane). Protein samples obtained from untransfected HeLa cells that had not been treated with chemicals were also subjected to 7.5% native PAGE. The separated proteins were transferred to PVDF membranes (Clearblot P™; ATTO Corporation, Tokyo, Japan). The membranes were blocked with Blocking One solution (Nacalai Tesque, Inc., Kyoto, Japan), before they were incubated with anti-Luc goat antibody (1∶10,000; Chemicon International, Inc., Temecula, CA, USA) or anti-β-gal rabbit antibody (1∶10,000; Chemicon International, Inc.) for 60 min. The membranes were then incubated for 30 min with horseradish peroxidase (HRP)-labeled anti-goat IgG or HRP-labeled anti-rabbit IgG (1∶10,000; Jackson ImmunoResearch Laboratories, Inc., PA, USA). Finally, each membrane was incubated with Amersham™ ECL Western Blotting detection reagent (GE Healthcare UK Ltd., Buckinghamshire, UK), after which the membranes were exposed to Polaroid film (FP-3000B, Fujifilm). The gray value of each specific band was measured using an image analysis program, as described above.

### *In vitro* inhibition assay using recombinant Luc

F-53 or PTC124 diluted with PLB (final concentration: 0.1, 1, or 10 µM) was added to recombinant Luc enzyme standard (obtained from *Escherichia coli*, 10 µg/mL: 0.16 µM, TOYO INK Co., Ltd.). Recombinant Luc enzyme standard was also diluted with PLB or HeLa cell lysate in PLB at 100 ng/mL to a final concentration of 1.6 nM. The Luc activity of these dilutions was then determined by measuring chemiluminescence on a GENios instrument after addition of PicaGene LT 2.0 (including the enzyme substrate, viz., luciferin, at a concentration of 470 µM).

### MALDI-TOF-MS and MS/MS analyses

Luc protein was purified using an anti-FLAG M2 affinity gel (Sigma-Aldrich Japan Inc) in the following manner: FreeStyle™293 cells were transfected with pCMV-Tag 1 control vector according to the instructions in the FreeStyle™293 Expression System manual (Life Technologies Japan Ltd). After 48 h, cells were treated with F-53 (10^−6^ M) or DMSO for an additional 20–24 h. After centrifugation, lysis buffer (50 mM Tris-HCl, pH 7.5; 150 mM NaCl; 0.5 mM EDTA; 0.1 mM ethylene glycol tetraacetic acid [EGTA]; 10% glycerol; 0.05% NP-40; 1 mM phenylmethylsulfonyl fluoride; and 50 mM sodium fluoride) was added to each cell pellet, which was then crushed using ultrasonic waves, before centrifugation (12,000×*g*, 30 min, 4°C). Each supernatant was applied to an anti-FLAG M2 affinity gel, after which the gel was washed with lysis buffer. Luc protein was obtained by elution with 100 µg/mL FLAG peptide in 50 mM Tris-HCl (pH 7.5), 150 mM NaCl, 0.5 mM EDTA, 0.1 mM EGTA, and 10% glycerol.

To compare the total masses of Luc protein from F-53-treated and untreated cells, samples containing 0.1% TFA were desalted using reverse-phase C4 ZipTips (Millipore Co., Billerica, MA, USA) and eluted with 50% CH_3_CN/0.1% TFA onto a stainless steel MALDI sample plate. Analysis was performed using an Autoflex III MALDI-TOF/TOF apparatus with CompassTM softwear (Bruker Daltonics K.K., Kanagawa, Japan) and a sinapinic acid matrix (Bruker Daltonics K.K.). The data were acquired in a positive linear mode of operation. To compare peptide mass data between F-53-treated and untreated Luc protein, samples were separated by 12% SDS-PAGE, followed by in-gel digestion. The discrete band corresponding to Luc (61 kDa) was excised from the Coomassie brilliant blue-stained gel and was then destained with 50% CH_3_CN in 25 mM ammonium bicarbonate (ABM, Fluka, Sigma-Aldrich Japan Inc). The destained gel section was reduced by treatment with 10 mM Tris(2-carboxyethyl)phosphine hydrochloride (Hampton Research Co., Aliso Viejo, CA, USA) dissolved in 25 mM ABM at 60°C for 10 min, and then alkylated by treatment with 55 mM iodoacetamide (Wako Pure Chemical Industries Ltd., Osaka, Japan) dissolved in 25 mM ABM in darkness at room temperature for 60 min. The gel section was washed with 50% CH_3_CN in 25 mM ABM and dehydrated. Trypsin (75 µL, 200 ng/µL, APRO Life Science Institute Inc., Tokushima, Japan) was added to the gel section, and the digestion reaction was allowed to proceed in darkness at 37°C overnight. TFA was added to the separated digestion reaction solution from the gel section to a final concentration of 0.1% TFA. The extracted peptides were desalted using C18 ZipTips (Millipore) and eluted with 50% CH_3_CN/0.1% TFA. Analysis was carried out as described above for the detection of the total mass of Luc protein by using a α-cyano-4-hydroxycinnamic acid matrix (Bruker Daltonics K.K.). The synthetic peptides GLTGKLDAR and GLTGK(F-53)LDAR were treated similar to the extracted peptides after digestion with trypsin.

### *In vivo* metabolism and *in vitro* formation of the CoA form of F-53

For *in vivo* observation of the metabolism of F-53 in mice, F-53 was suspended in 0.5% methyl cellulose solution and administered to mice (ICR mice, male, 6 weeks old) by oral intubation (3 mg·kg^−1^·d^−1^). After 1 h, blood was collected from the inferior vena cava of the anesthetized mouse and centrifuged at 13,700×*g* (4°C, 5 min). MeOH was added to and mixed with the plasma; then, the samples were centrifuged at 13,700×*g* (20°C, 5 min), and the supernatant was analyzed by HPLC/MS, using the system and analytical method described for the synthesis of F-53-CoA.

*In vitro* formation of F-53-CoA was initiated by addition of 1.44 µg QuantiLum® Recombinant Luc (0.133 µM, Promega) or mouse liver microsomes (27.6 µg) into 2.5–100 µM F-53 solution (50 mM Tris-HCl, pH 7.5; 10 mM MgCl_2_; 0.2 mM DTT; 2 mM ATP; 2 mM CoA; 0.1% Triton X-100). Mouse liver microsomes were prepared as described previously [26] were provided by Hoshi University (Tokyo, Japan). The reaction mixture was incubated for 15 or 20 min at 37°C. The reactions were terminated by the addition of ice-cold CH_3_CN. After centrifugation at 14,000×*g* and 4°C for 10 min, the supernatant was analyzed by the Waters HPLC/UV and HPLC/MS systems by using the analytical method described for F-53-CoA synthesis. The assays were performed in triplicate with F-53 (2.5–100 µM) as the substrate. The K_m_ and V_max_ values for F-53 were determined at concentrations ranging from 2.5 to 100 µM, by using Lineweaver-Burk plots.

### Statistical analysis

Each value has been expressed in terms of the mean±standard error of the mean (SEM). The dose-response curve of F-53 for the hRORα1 assay with the Luc reporter was generated using the scientific graphing and analysis software Origin (version 7.5; OriginLab Co., Northampton, MA, USA). Statistical analysis was performed using the Excel statistics 2010 software (Social Survey Research Information Co. Ltd, Tokyo, Japan). Statistical differences in reporter enzymatic activities, *Luc* mRNA, and protein levels were determined by one-way factorial ANOVA and post-hoc analysis with Fisher's LSD test. P<0.05 was considered statistically significant.

## Results

### Dose response of F-53 inhibition

**Figure 2:**
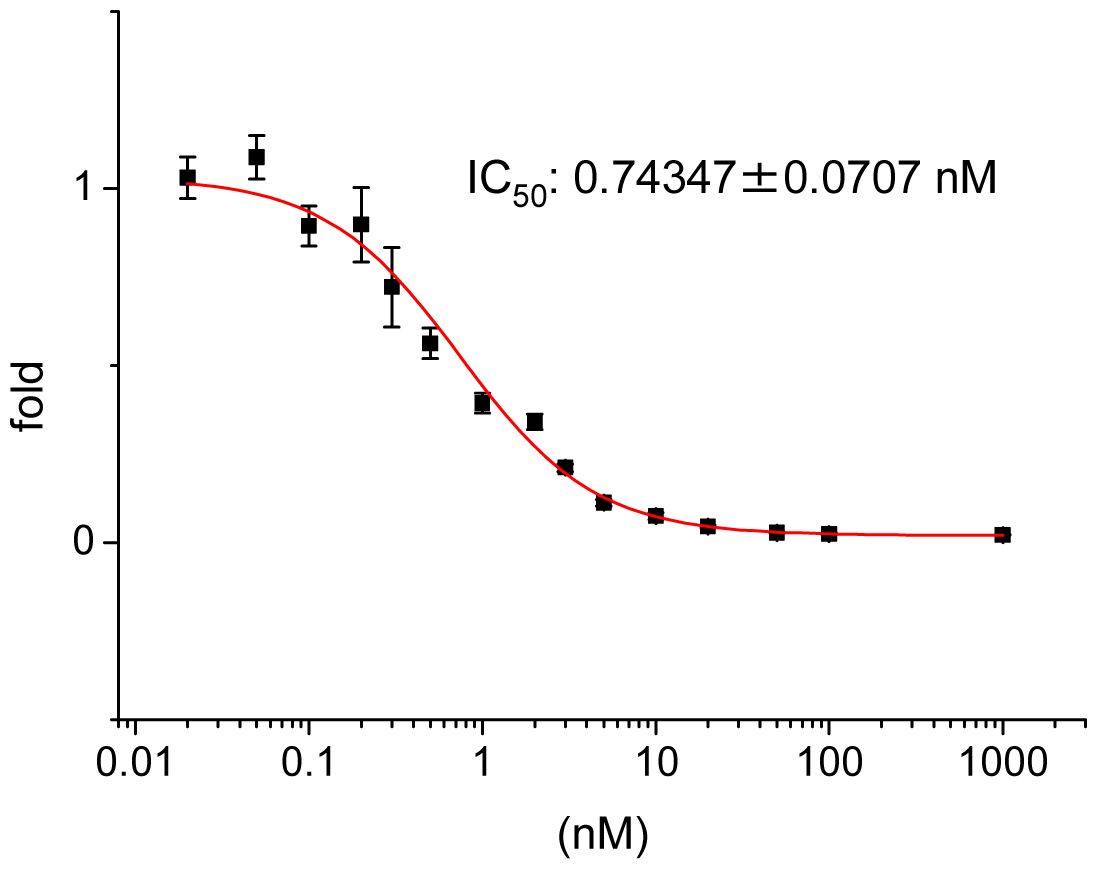
Inhibition of firefly luciferase (Luc) activity by F-53, as determined using a reporter assay. The dose-response curve for F-53 was constructed by using the human retinoid orphan receptor α1 (hRORα1) reporter assay. Mammalian expression vectors for full-length hRORα1 (pcDNA3-RORα1), thymidine kinase promoter-based Luc reporter, (Rev-DR2×3-TK-Luc), and pCMVβ were transfected into HeLa cells. The cells were treated with F-53 (10^−6^–2×10^−11^ M) for 1 d. Luc activity was determined after the addition of PicaGene LT 2.0 and normalized relative to β-gal activity. The results (Y-axes) are expressed as the fold increase in Luc activity after F-53 treatment, relative to the increase after treatment with DMSO. Each value represents the mean±SEM of six transfection experiments.

F-53 was synthesized as the ligand for nuclear receptors for use in the ligand screening assay, which used Luc as the reporter. Given that preliminary ligand screens indicated the ability of F-53 to substantially inhibit hRORα1, we used the hRORα1 reporter assay to construct a dose-response curve for F-53. The dose-response curve indicated that the 50% inhibitory concentration (IC_50_) of F-53 was 0.743±0.0707 nM (Figure 2).

### Specificity of F-53 inhibition

**Figure 3:**
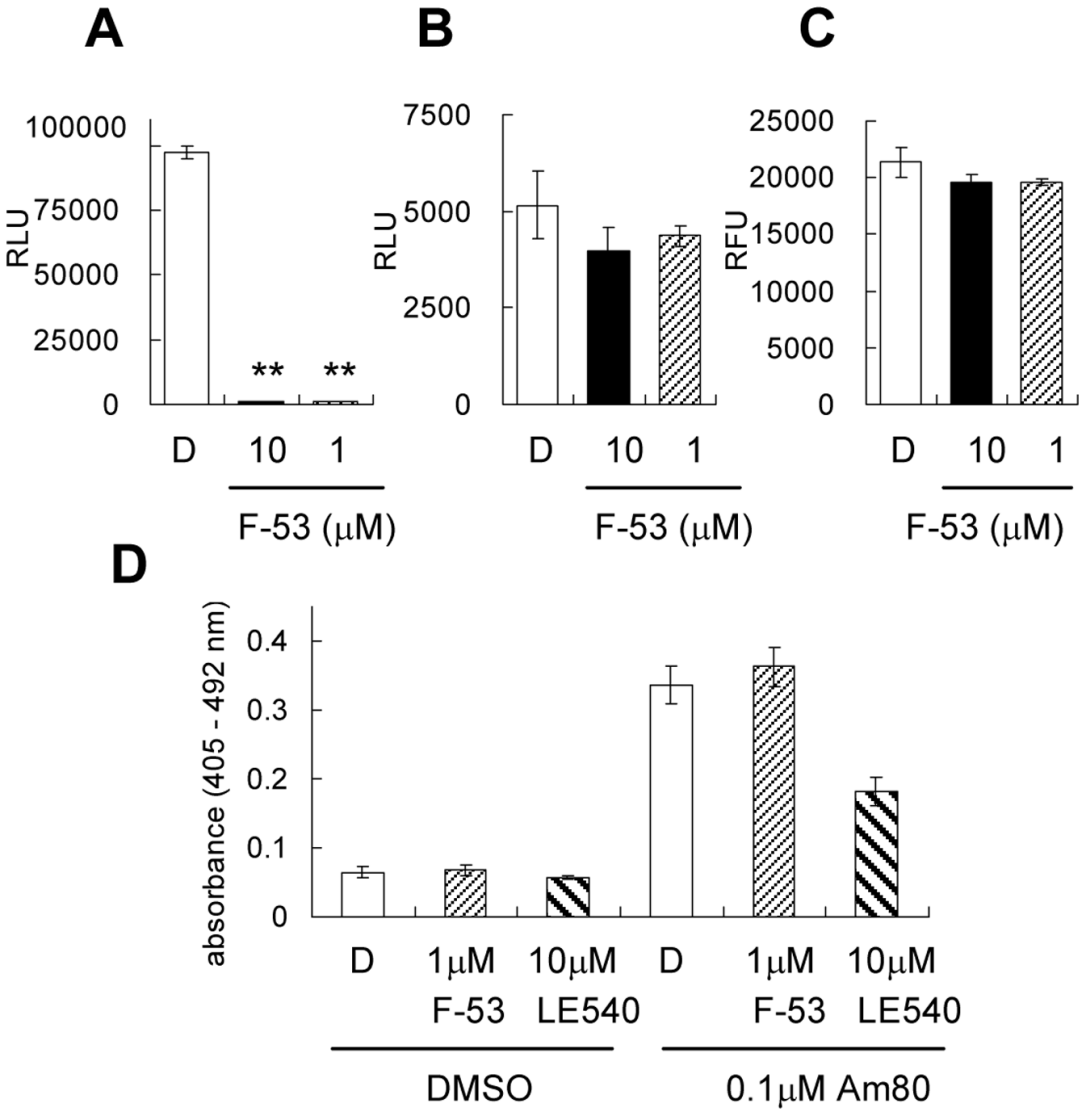
Effect of F-53 on reporter enzymes. The effects of F-53 on firefly luciferase (A), Renilla luciferase (B), β-galactosidase (β-gal) (C), and chloramphenicol acetyl transferase (CAT) (D) are shown. A–C: All individual reporter plasmids, that is, pCMVluc+ (A), pRL-CMV (B), and pCMVβ (C), were transfected into HeLa cells. The relevant enzymatic activity in cell lysates was measured after treatment with DMSO or F-53 (1 and 10 µM), in the presence of the relevant enzymatic substrate. D: pSG5-hRARα1 DR5G-TK-CAT, and pCMVβ were transfected into COS-1 cells. After treatment with DMSO, F-53, and LE540 (RAR pan-antagonist) with or without Am80 (RARα,β-selective agonist), CAT activity in cell lysates was measured by a CAT ELISA, and the results were normalized relative to β-gal activity. The asterisks indicate statistically significant differences (** p<0.01) between F-53 and DMSO treatments, which were determined using post-hoc analysis with Fisher's LSD test. D: DMSO, CAT: chloramphenicol acetyl transferase, RLU: relative light unit, RFU: relative fluorescence unit. Each value represents the mean±SEM of three transfection experiments.

To confirm the specificity of F-53 inhibition, Luc activity was determined in cells that had been transiently transfected with the CMV-Luc plasmid, in which the expression of the Luc gene is not under the control of the response element for hRORα1. However, Luc activity was significantly reduced by treatment with 1 µM F-53 (Figure 3A). We then used transient expression assays to investigate the likelihood that F-53 and other ligands tested in the screen might directly affect the activities of reporter proteins. Treatment with 1 µM F-53 decreased RL activity slightly (Figure 3B) but had no effect on β-gal activity (Figure 3C) when these reporter genes were expressed without regulation by the hRORα1 response element. When placed under the control of the RARα response element, namely, the ligand screening assay for RARα using CAT as the reporter, CAT activity increased following treatment with a RARαβ-selective agonist (0.1 µM Am80) and the increasing activity was inhibited by treatment with a RAR pan-antagonist (10 µM LE540), whereas treatment with 1 µM F-53 showed no effect (Figure 3D). However, 1 µM F-53 showed inhibition in the results using a Luc reporter under the control of the RARα response element (data not shown), with results comparable to those shown in Figure 3A. Therefore, we deduced that the inhibitory effect of F-53 resulted not from an effect on transcriptional activity, but from a specific effect on Luc activity.

### Time course of changes in Luc activity

**Figure 4:**
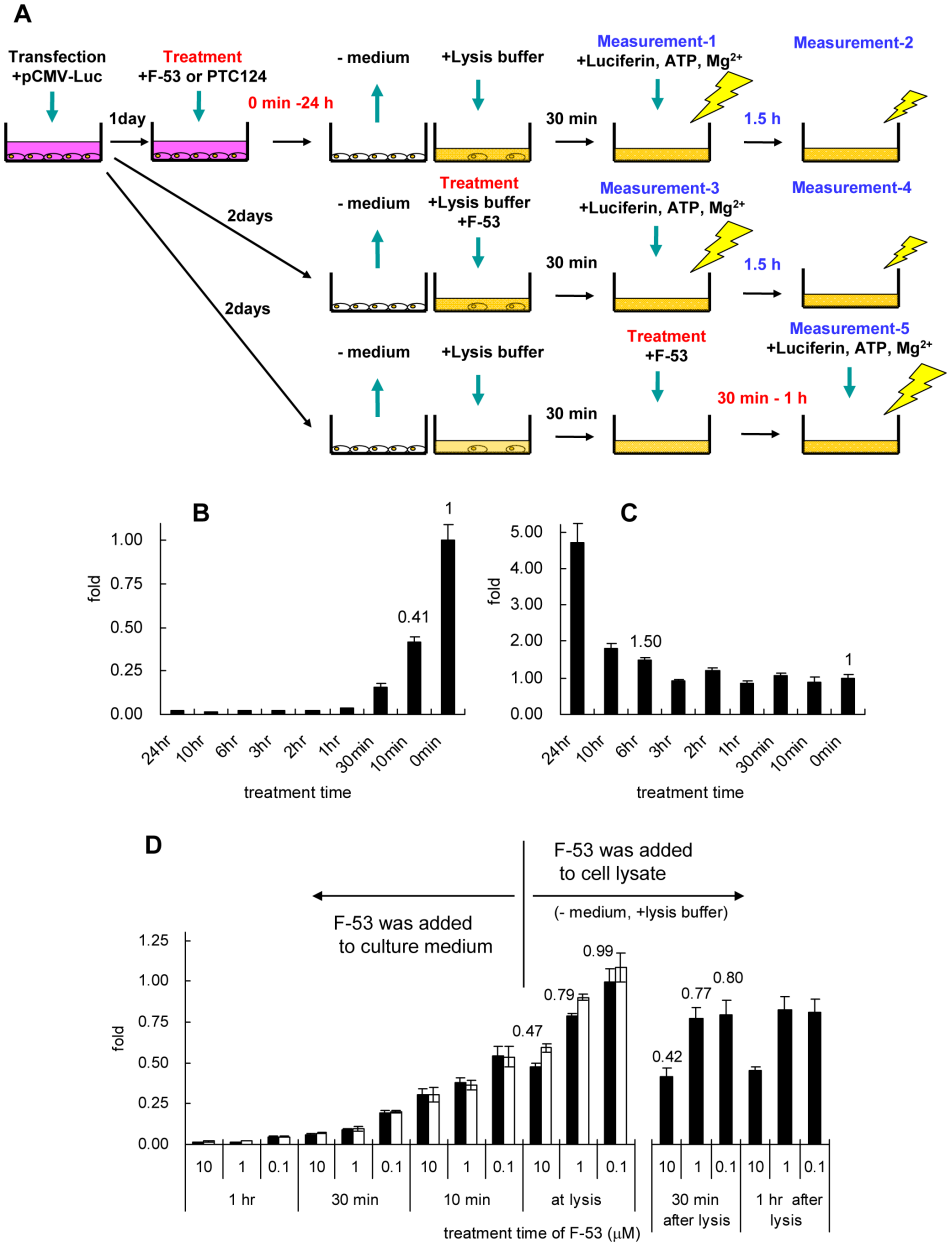
Time course of changes in firefly luciferase (Luc) activities. A: Protocol for treating cells and measuring Luc activity. HeLa cells were transiently transfected with the pCMVluc+ and the pCMVβ to normalize differences in transfection efficiency. The cells were then treated with F-53 (B: 1 µM, D: 0.1–10 µM,) or PTC124 (C: 0.1 µM) for the incubation periods indicated in the figure (B–C). B and C: Luc activity was determined by measurement-1 in (A). D: Luc activity after the addition of F-53 in culture medium was determined by measurement-1 (black bar) and measurement-2 (white bar) in (A). Luc activity after the addition of F-53 at the same time as lysis was determined by measurement-3 (black bar) and measurement-4 (white bar) in (A). Luc activity after the addition of F-53 after lysis was determined by measurement-5 in (A). Treatment with F-53 after cell lysis involved treatment with a solution of F-53 in lysis buffer solution. Luc activity was normalized relative to β-gal activity. The results (Y-axes) are expressed as the fold increase in Luc activity after F-53 or PTC124 treatment, relative to the increase after treatment with DMSO. Each value represents the mean±SEM of three transfection experiments.

To clarify the inhibition profile of F-53 for Luc enzymatic activity, Luc activity was measured after treatment with F-53 for different periods. The time course of changes in Luc activity in transiently transfected HeLa cells after treatment with F-53 or PTC124 is shown in Figure 4. Treatment with F-53 in the cell culture medium (1 µM) markedly inhibited Luc activity within 1 h, and this level of inhibition was sustained for 24 h (Figure 4B). PTC124 (0.1 µM) had no effect on Luc activity within 3 h; however, a 4.72-fold increase in activity after 24 h (Figure 4C) suggested that PTC124 and F-53 had different effects on Luc activity in cells. The inhibitory effect of F-53 decreased rapidly after cell lysis, and the level of inhibition following treatment with F-53 after lysis remained constant for at least 1 h after lysis (Figure 4D, black bar). The Luc activity in the cells treated with F-53 at any later time point (Figure 4D, black bar) was comparable to the activity at 1.5 h after lysis (Figure 4D, white bar).

### *Luc* mRNA and protein levels in cells after treatment with F-53

**Figure 5:**
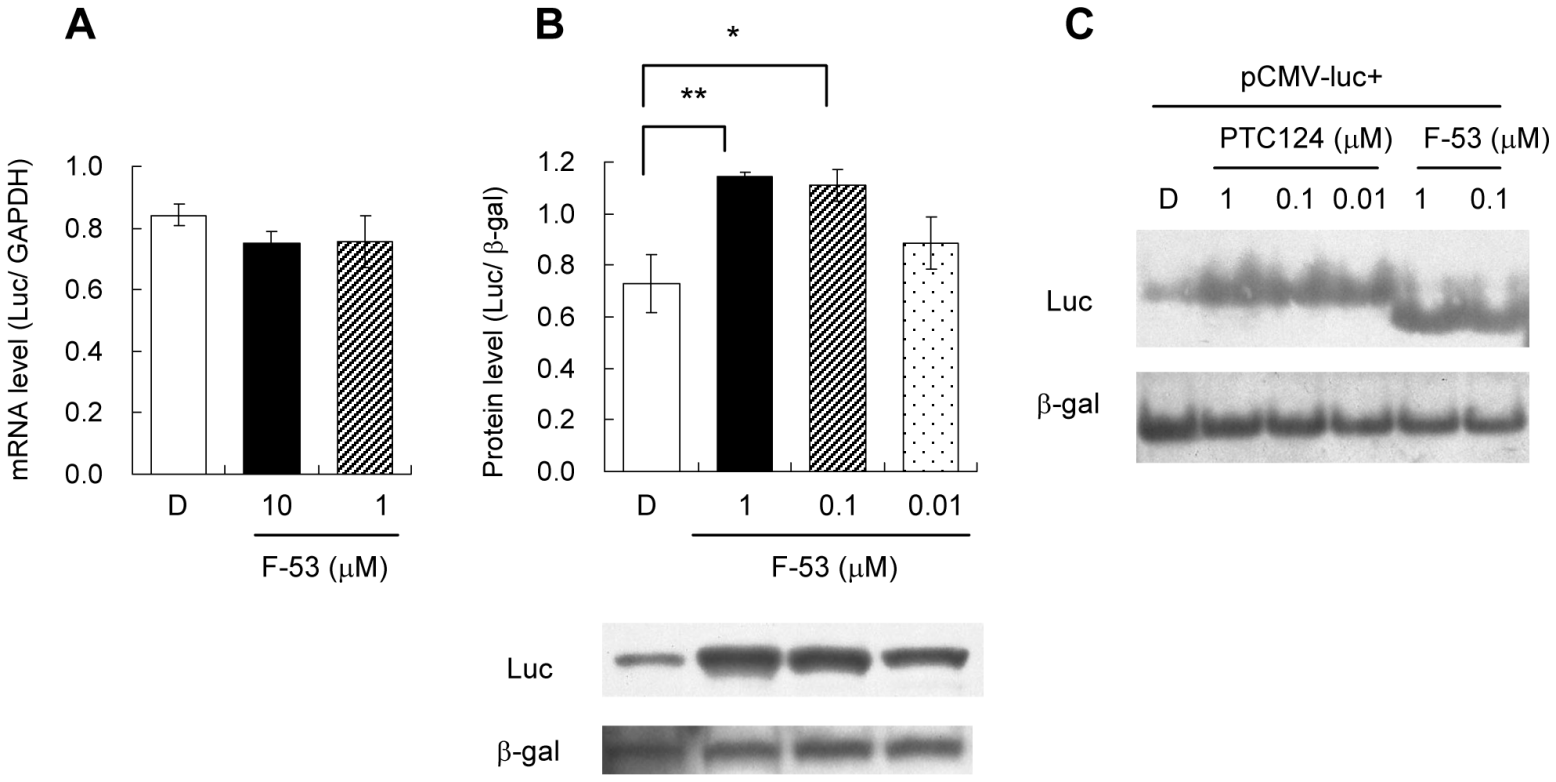
Effects of F-53 on levels of firefly luciferase (*Luc*) mRNA and protein in culture medium. A: *Luc* mRNA expression was determined by RT-PCR. Total RNA was isolated from HeLa cells transiently transfected with pCMVluc+ and treated with F-53 or DMSO for 20–24 h. The expression of *Luc* was normalized relative to the abundance of the endogenous *hGAPDH* transcripts. B and C: Western blotting after SDS-PAGE (B) and native PAGE (C). Protein samples for SDS- or native PAGE and western blot analysis were obtained from HeLa cells that had been transiently transfected with pCMVluc+ and pCMVβ and treated with F-53, PTC124, or DMSO for 20–24 h before lysis. The protein level of Luc was normalized relative to that of β-gal. The asterisks indicate statistically significant differences (* p<0.05, ** p<0.01) between F-53 and DMSO treatments, determined using post-hoc analysis with Fisher's LSD test. D: DMSO, Luc: Luciferase. Each value represents the mean±SEM of three independent experiments.

The effect of F-53 on *Luc* mRNA expression was determined by RT-PCR (Figure 5A). Comparison with levels of *hGADPH* mRNA (endogenous control) indicated that F-53 did not significantly affect *Luc* mRNA levels.

Western blotting after SDS-PAGE showed that treatment with F-53 induced a significant increase in Luc protein levels (Figure 5B) despite causing a rapid decrease in Luc enzymatic activity (Figure 4B). Moreover, inclusion of F-53 in the culture medium promoted the migration of Luc protein during native PAGE (Figure 5C). Inclusion of PTC124 in the culture medium did not affect migration of Luc during native PAGE but increased the level of Luc protein (Figure 5C).

### Effect of F-53 on the recombinant Luc standard *in vitro*

**Figure 6:**
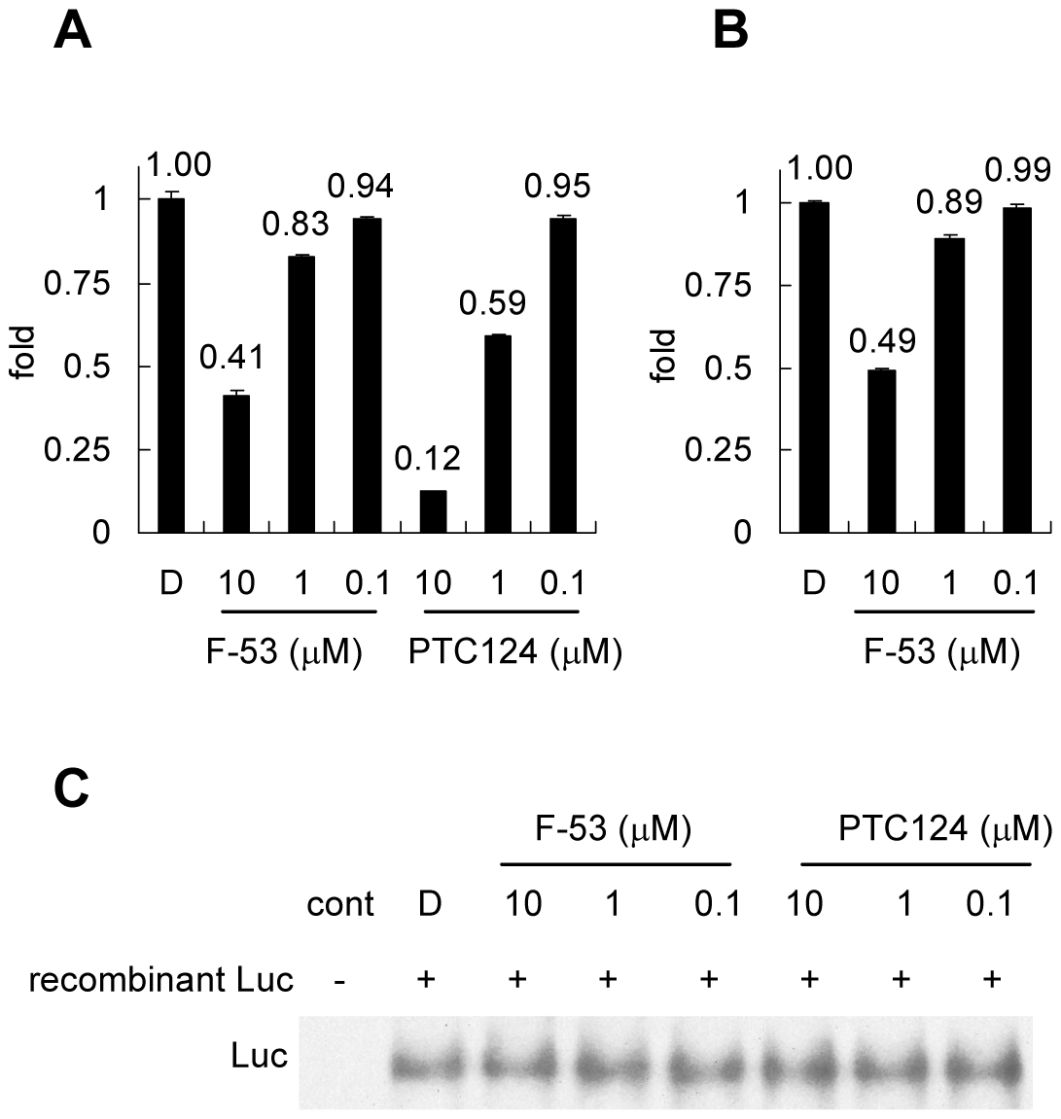
Effect of F-53 on recombinant luciferase (Luc) activity *in vitro*. The inhibitory effects of F-53 on recombinant Luc enzymatic activity in lysis buffer (A) and HeLa cell lysate (B) are shown. The fold increase (Y axes) in Luc activity after treatment with either F-53 or PTC124 was normalized relative to the level obtained after DMSO treatment. The values shown above the bars represent the mean, and the error bars indicate SEM of three independent experiments. C: Western blotting after native PAGE by using the Luc protein sample used in (A). We added protein samples for native PAGE (12.5 ng protein), obtained from HeLa cells, to each Luc protein sample used in (A) because the band representing recombinant Luc protein alone was not clearly visible on western blotting of native PAGE gels, and then used western blotting to analyze these proteins after native PAGE. Cont represents samples with 12.5 ng of HeLa protein only; no Luc band was detected in this control.

To clarify the difference in inhibition between treatment with F-53 in culture medium and F-53 treatment after lysis (Figure 4D), the inhibitory effect of F-53 on recombinant Luc enzymatic activity was confirmed using PLB and the substrate mix, PicaGene LT 2.0 (which includes the enzyme substrate, luciferin, at a concentration of 460 µM). Both reagents were obtained from the Luc reporter assay kits used. Figure 6 compares the fold change in the activity of recombinant Luc (100 ng/mL) after treatment with F-53 or PTC124 with that after treatment with DMSO. Treatment with 10 µM F-53 decreased Luc activity by less than 50% (Figure 6A). Treatment with 10 µM PTC124 caused greater inhibition of recombinant Luc than that achieved by F-53 (Figure 6A). The addition of HeLa cell lysate had no effect on the activity of recombinant Luc in this *in vitro* assay (Figure 6B). The recombinant Luc samples used in this *in vitro* inhibition assay (Figure 6A) were subsequently subjected to western blotting after native PAGE. However, we did not observe any change in the migration of Luc during native PAGE after *in vitro* treatment of recombinant Luc with either F-53 or PTC124 (Figure 6C).

### Modification of Luc protein in living cells by treatment with F-53

**Figure 7:**
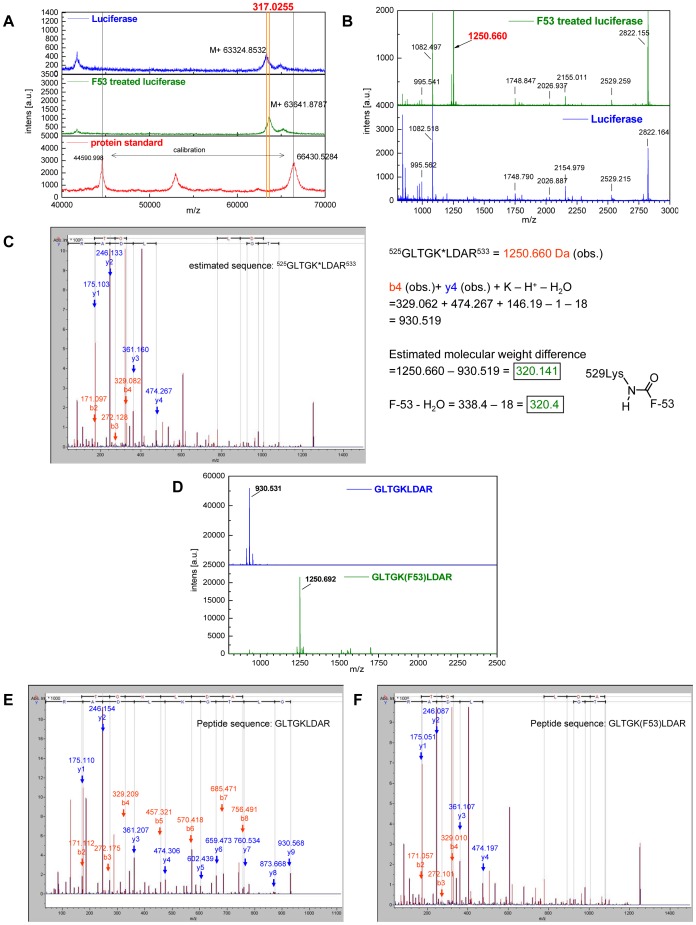
MALDI-TOF-MS and MS/MS analyses of firefly luciferase (Luc) protein modified by F-53 and synthetic peptides. A: Total masses of Luc proteins after F-53 treatment compared with those after the control treatment (DMSO). B: Peptide mass spectra of Luc proteins after F-53 (upper) and DMSO (lower) treatments. C: MS/MS analysis of the 1,250 Da peak in (B). MS/MS analysis estimated that the peptide sequence of 1,250 Da peak is ^525^GLTGK*LDAR^533^ which was not digested at the lysine-529 position by trypsin. The molecular weight (1,250 Da) was 320 Da greater than that calculated from the actual peptide sequence. The difference was attributable to the presence or absence of F-53 at the amino group of lysine-529. D: MALDI-TOF-MS analysis of the synthetic peptides GLTGKLDAR (upper) and GLTGK(F-53)LDAR (lower), in which the amino group of the lysine and the carboxyl group of F-53 were dehydration-condensed. GLTGK(F-53)LDAR is the estimated sequence in (C). E and F: MS/MS analysis of the synthetic peptides GLTGKLDAR (E) and GLTGK(F-53)LDAR (F). The MS/MS data of (C) were the same data as those of (F).

To analyze the effect of treatment with F-53 with respect to the physical alteration of Luc protein in living cells, MALDI-TOF-MS and MS/MS analyses were performed on Luc proteins purified from cells treated with or without F-53. Treatment with F-53 shifted the mass spectral peak of Luc protein; treatment of cells with F-53 reproducibly increased the peak for the Luc protein to approximately 317 Da more than that of Luc protein from untreated cells (Figure 7A).

The peptide mass spectra of trypsin-digested Luc proteins after treatment with F-53 are shown in Figure 7B. A peptide peak was confirmed at approximately 1,250 Da in the mass spectrum of Luc protein treated with F-53 (Figure 7B, upper). MS/MS analysis of the 1,250-Da peak (Figure 7C) showed the sequence of the corresponding peptide to be 525GLTGK*LDAR533. Trypsin did not digest this peptide at lysine-529. The molecular weight of this peptide (1,250 Da) was 320 Da greater than that calculated from the actual peptide sequence (930 Da); the difference in weight was related to the presence or absence of F-53, which we propose binds to the amino moiety of lysine-529 (Figure 7C).

Next, we synthesized the peptides GLTGKLDAR and GLTGK(F-53)LDAR, using the sequences estimated from the data shown in Figure 7C. MALDI-TOF-MS analysis of the synthetic peptides is shown in Figure 7D. The mass spectral peak of the peptide covalently bound to F-53 was confirmed at 1,250 Da (Figure 7D, lower). MS/MS analyses of the synthetic peptides GLTGKLDAR and GLTGK(F-53)LDAR are shown in Figures 7E and F. The MS/MS data in Figure 7C were identified as being equivalent to those shown in Figure 7F. Eventually, using the synthetic peptide, the peptide sequence after trypsinization was estimated to be GLTGK(F-53)LDAR (Figure 7F).

### Mutation at lysine-529

We inserted a mutation at lysine-529 to confirm that this residue is required for enzymatic activity of Luc. After they had been normalized relative to β-gal activity, Luc activities in the presence of mutation of lysine-529 to either alanine, glutamate, or arginine were 31±3, 26±1, and 37±3 relative light units (RLUs, mean±SEM), respectively, compared with the native Luc activity, which was 15,998±2,253 RLU (n = 6 for all). The absence of enzymatic activity of the K529A, K529E, and K529R mutants suggests that lysine-529 is crucial for the enzymatic activity of Luc.

### Production of F-53-CoA by using Luc and microsomes

**Figure 8:**
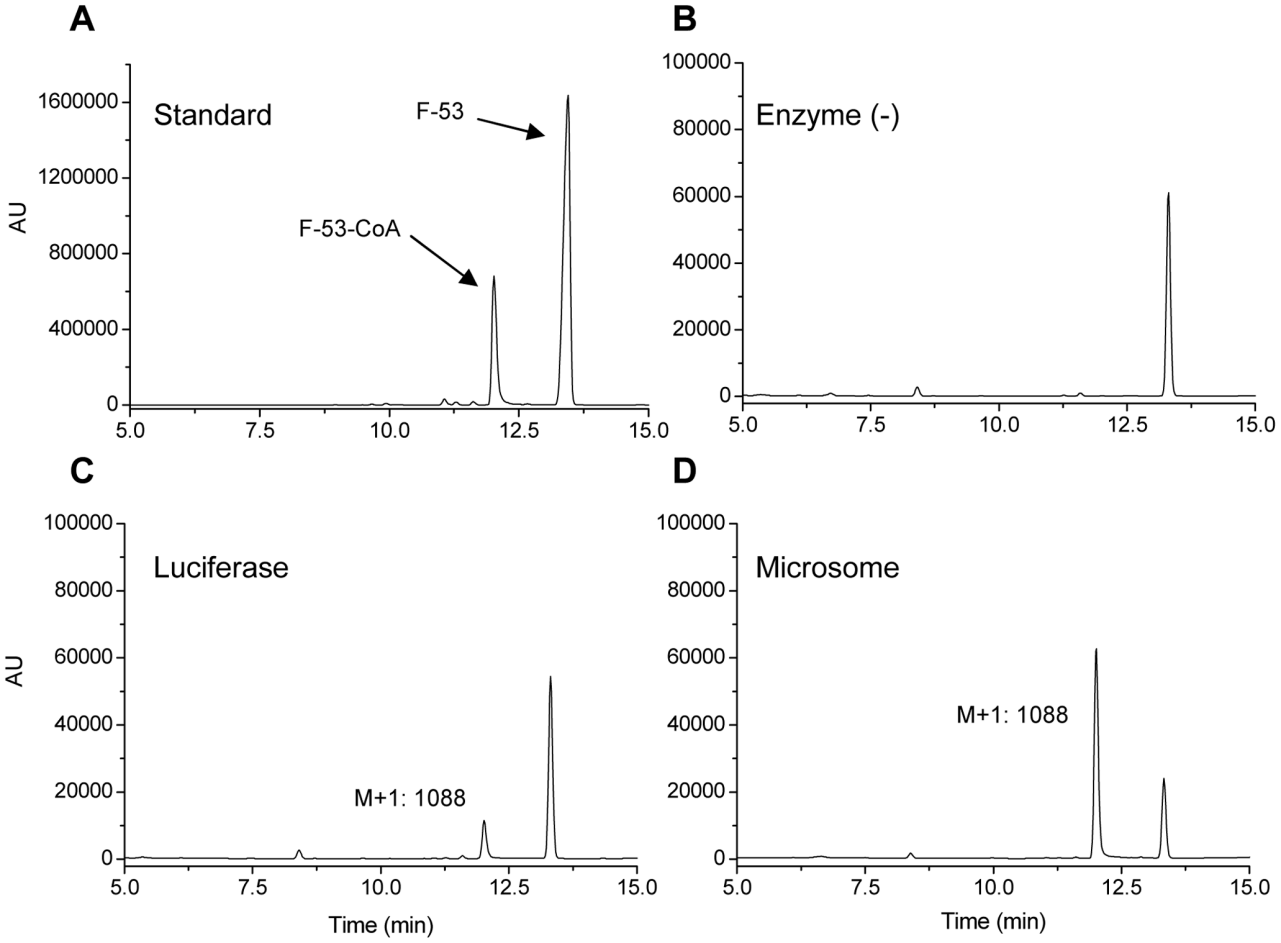
*In vitro* production of F-53-CoA. The typical HPLC/UV chromatograms obtained from standard mixture (A), the assay samples without enzyme (negative control, B), by using recombinant luciferase (0.133 µM, C), and by using mouse liver microsomes (27.6 µg, D) with ATP, MgCl_2_, and CoA-SH are shown. Enzymatic *in vitro* assays were performed at 37°C for 15 min with F-53 (20 µM) as the substrate. The formation of F-53-CoA (product) was measured by HPLC/UV at 260 nm by using 10%–90% CH_3_CN in water with 10 mM AcONH_4_ (pH 7.0) as the mobile phase. Mass analysis was performed by HPLC/MS (positive-mode ESI) using the same mobile phase.

To investigate the mechanism by which F-53 reacts with lysine-529 of Luc, we tested whether recombinant Luc and mouse liver microsomes are able to produce F-53-CoA *in vitro*. We did not directly demonstrate the presence of an F-53-AMP adduct in the present study. If lysine-529 of Luc is acetylated by acetyl-CoA, it seems likely that F-53 also needs to be activated to F-53-CoA in cells. The motivation for using mouse liver microsomes was that we had previously detected a metabolite of F-53 produced by β-oxidation, which was produced from F-53-CoA in the plasma of a mouse given F-53 (3 mg·kg^−1^·d^−1^) orally (data not shown). The typical HPLC/UV chromatograms obtained from *in vitro* assay samples are shown in Figure 8. The peak with the same molecular weight as F-53-CoA (M+1: 1088) appeared at 12 min in chromatograms obtained from *in vitro* assays of both recombinant Luc (Figure 8C) and mouse liver microsomes (Figure 8D), and was identified using synthetic F-53-CoA as the standard (Figure 8A). Furthermore, the kinetic parameters K_m_ and V_max_ were calculated for Luc and microsomes in triplicate with F-53 (2.5–100 µM) as the substrate, although the values of these kinetic parameters were not reflected precisely in the enzymatic activities. The affinity of Luc for F-53 (K_m_ = 1.61 µM) was greater than that of the microsomes (K_m_ = 76.2 µM), and the reaction velocity of Luc for F-53 (V_max_ = 82.0 nmol·min^−1^·mg^−1^ protein) was more than 5-fold greater than that of the microsomes (V_max_ = 14.8 nmol·min^−1^·mg^−1^ protein).

### Effect of kinase inhibitors on Luc modification by F-53

**Figure 9:**
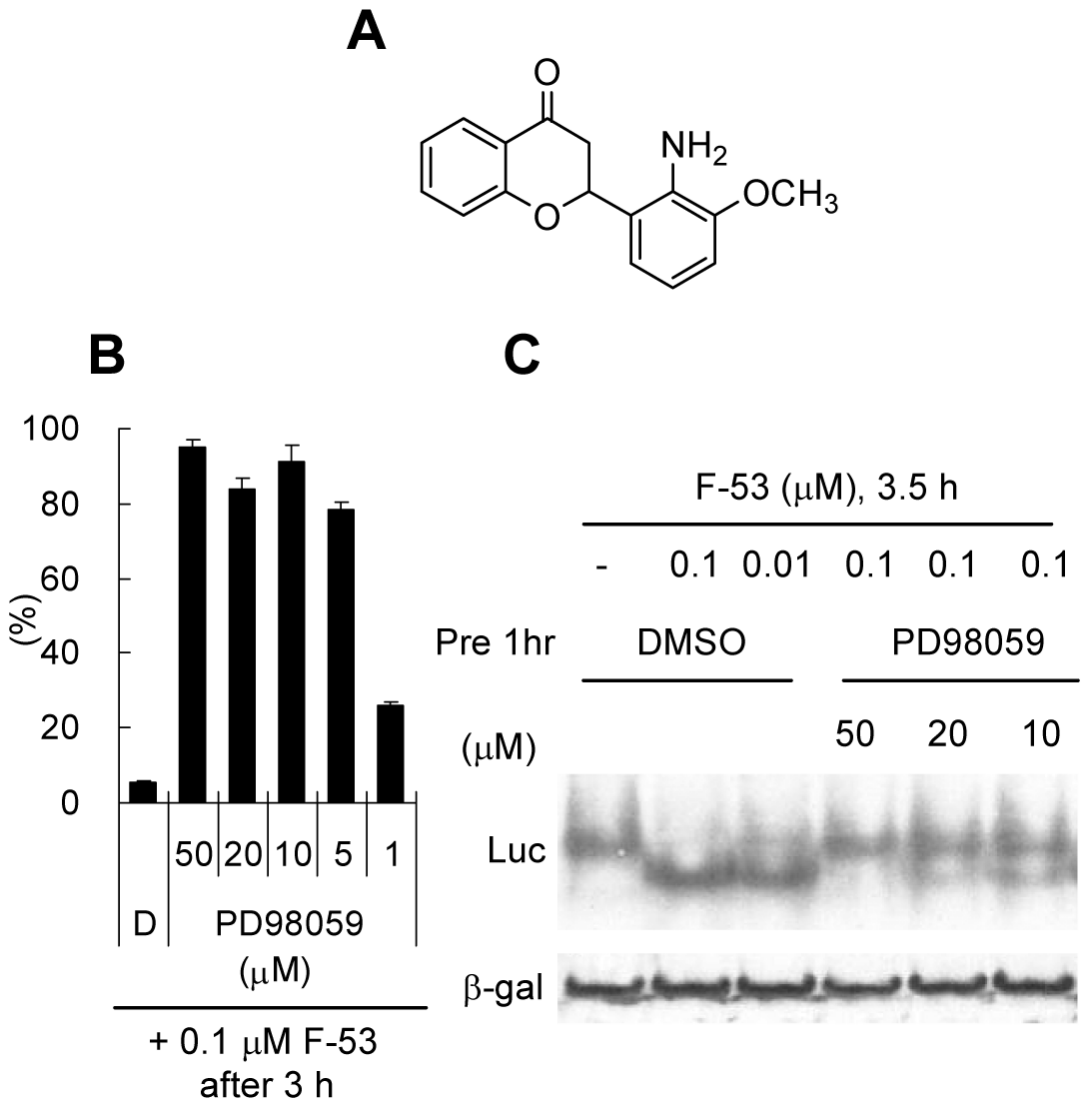
Effect of a kinase inhibitor on firefly luciferase (Luc) modification by F-53. A: The structure of PD98059. B: Effect of PD98059, a mitogen-activated protein kinase kinase (MEK) inhibitor for F-53 inhibition. PD98059 was added to HeLa cells transiently transfected with pCMVluc+ and pCMVβ to normalize differences in transfection efficiency at 3 h prior to the addition of F-53 (0.1 µM). Luc activity was determined after the addition of PicaGene LT 2.0 and normalized relative to β-gal activity. The results (Y-axes) are shown as the percentage of Luc activity relative to the DMSO control. C: Western blotting after native PAGE. PD98059 was added to HeLa cells transiently transfected with pCMVluc+ and pCMVβ at 1 h prior to F-53 or DMSO treatment for 3.5 h. HeLa cells lysates were subjected to native PAGE and western blotting. Luc: Luciferase. Each value represents the mean±SEM of three transfection experiments.

We tested the effects of several kinase inhibitors on Luc enzymatic activity to investigate whether kinases are involved in the mechanisms that underlie Luc modification by F-53 (data not shown). Treatment with 5 µM PD98059 (Figure 9A), which is reported to function as a mitogen-activated protein kinase kinase (MEK) inhibitor [27], restored over 80% of Luc activity when Luc activity was inhibited in the presence of 0.1 µM F-53 (Figure 9B). The observation that treatment with 50 µM PD98059 prevented the F-53-induced shift in the migration of Luc protein after native PAGE (Figure 9C) is consistent with the conclusion that PD98059 abrogates F-53-mediated inhibition of Luc enzyme activity. However, 100 µM PD98059 did not inhibit Luc activity or show toxicity in HeLa cells after a single treatment (data not shown).

## Discussion

We firstly investigated the inhibition mechanism of F-53 for Luc activity. Both the MALDI-TOF-MS and MS/MS results demonstrated that the protein modification of Luc by F-53 in living cells entailed acylation of lysine-529 with the carboxyl group of F-53 (Figure 7). This seemingly small modification had unexpected consequences. Branchini *et al.* performed a detailed kinetic analysis using Luc with mutation of lysine-529 to either alanine, glutamine, or arginine, as part of a study of the active site of Luc; they showed that lysine-529 is a critical residue for effective substrate orientation and that it provides important favorable polar interactions [28]. We also demonstrated the importance of lysine-529 for Luc activity in this study: modification of lysine-529 to alanine, glutamate, or arginine completely abolished enzymatic activity (see the **Mutation at lysine-529** subsection in the Results section). Starai *et al.* proposed that acetylation of lysine-529 modulates Luc activity [8]. Indeed, lysine acetylation and deacetylation constitute an important enzyme-switching mechanism; for instance, histone acetylation induces transcription [10]. The F-53 adduct of lysine-529 probably abolished Luc activity by changing the conformation of Luc (Figure 5C), possibly by blocking the flexible conformation change that mediates the first half of the Luc reaction, which involved adenylation by ACSL [7], [8], [10], [29].

We next investigated the mechanism underlying the reaction between F-53 and lysine-529 of Luc in cells. We hypothesized that F-53 was activated by conjugation with CoA and that the F-53-CoA that was formed then reacted with lysine-529 of Luc. This proposal was based on the suggestion that lysine-529 of Luc forms an adduct with acetyl-CoA during the step that normally results in the acetylation of Luc. Luc might employ its ACSL activity to activate F-53-CoA [5], [6]. However, the metabolism of F-53 by β-oxidation in mammals demonstrated that F-53 was activated to F-53-CoA in mammals in the absence of Luc. We considered that differences in the activities of recombinant Luc and other enzymes that metabolize F-53 in mammals might help to clarify the ligation activity of F-53 in mammals. Therefore, we used mouse liver microsomes and recombinant Luc to assess Luc activity *in vitro* in the presence of ATP, Mg^2+^, and CoA-SH (Figure 8). The comparison with kinetic parameters of recombinant Luc and mouse liver microsomes showed that Luc easily catalyzed the conversion of F-53 to F-53-CoA.

High levels of acetyl-CoA may directly cause lysine acetylation, which would account for the small number of cytoplasmic and mitochondrial protein acetyltransferases involved in metabolic regulation [10]. We suggest that some mammalian protein transferases might contribute to the transfer of F-53-CoA to lysine-529. The first reason is that although there are many lysine residues in the proximity of lysine-529 in Luc (e.g., lysine-524, lysine-534, lysine-541, lysine-543, and lysine-544), F-53 is ligated exclusively to lysine-529 (Figure 7). The second reason is that Luc is inactivated in cells before their lysis (Figure 4D); this might occur either because the lysis buffer deactivates the transferase involved in the transfer of F-53-CoA or because the membrane structure in a living cell is necessary for the transfer of F-53-CoA to lysine-529. Indeed, ACSL dimers attach to the membrane, where they metabolize and transport substrate [29]. Furthermore, PD98059, which is reported to function as an MEK inhibitor, might prevent the acylation of lysine with F-53 in a concentration-dependent manner (Figure 9). PD98059 exerts its inhibitory effect by binding to MEK kinase, thus blocking the signaling cascade downstream of MEK activation [27]. In this regard, recent studies have demonstrated that phosphorylation was required before acetylation in some case; AMP-activated protein kinase promotes p53 acetylation via phosphorylation [30], and the acetylation of NK-κB p65 at lysine-310 is inhibited by blocking phosphorylation of serine-276 [31]. An alternative explain is that PD98059 might simply block luciferase access to F53-CoA instead affecting a regulatory phosphorylation event; this would be consistent with the weak inhibitory effect of PD98059 on the enzyme activity of Luc [32]. However, the enzyme responsible for the conjugation of F-53 to lysine remains to be clarified.

**Figure 10:**
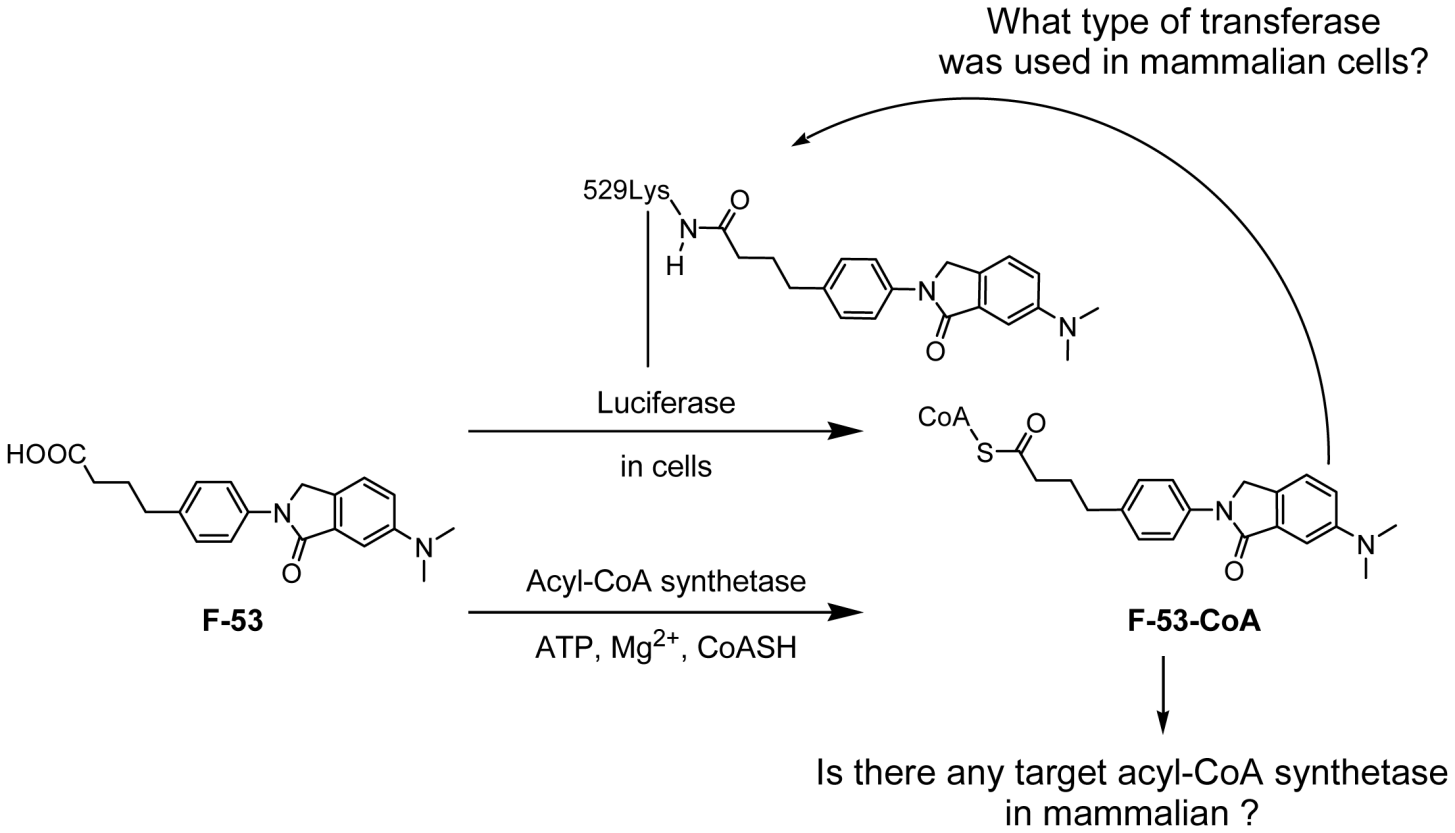
Proposed mechanism for the reaction of F-53 with firefly luciferase (Luc). The mechanism might involve F-53 activation to its CoA-derivative by Luc, which functions as an acyl-CoA synthetase in living cells. F-53-CoA is proposed to be transferred to lysine-529 of Luc by an unknown cellular acetyltransferase. Lysine-529 might be the most important lysine for the regulation of enzyme activity by acetylation and deacetylation. We also suggest that after F-53-CoA is activated by native acyl-CoA synthetase in mammalian liver microsomes, F-53-CoA may be transferred to the lysine residue, which induces the acetylation of the same enzyme.

Taken together, our data suggest that F-53 is metabolized to F-53-CoA by Luc and that a mammalian transferase transfers activated F-53-CoA to lysine-529 of Luc, which inactivates Luc (Figure 10).

Our data further demonstrated that F-53 inhibited Luc only slightly after cell lysis and that the level of inhibition did not change after either shortening or prolonging the duration of treatment with F-53 (Figure 4D). F-53 showed the same level of inhibition of Luc in a cell-free *in vitro* assay involving recombinant Luc as was observed in cells; however, whereas PTC124 caused greater inhibition than F-53 in the cell-free *in vitro* assay (Figure 6A), inclusion of PTC124 in culture medium failed to inhibit Luc activity in cells (Figure 4C). Auld *et al.* demonstrated that inhibition by PTC124 is the result of an inhibitory product formed during the Luc-catalyzed reaction between ATP and PTC124 [12],[13]. The reaction with ATP is the first step both in the oxidation of the Luc substrate luciferin and in the synthetic activation of acyl-CoA [5]. Therefore, this inhibition of Luc by F-53 without a change in protein structure evident after native PAGE (Figures 5C and 6C) suggested that F-53 might inhibit the initial oxidation of luciferin.

One of the unresolved issues that emerged from this study is the demonstration that treatment with F-53 increased the levels of Luc protein in living cells (Figures 5B and C). We think that it is unlikely that this increase resulted from increased biosynthesis; instead, we propose that it resulted from delayed degradation of Luc protein, because treatment with F-53 failed to affect the level of *Luc* mRNA (Figure 5A). Moreover, the PTC124-mediated increase in Luc activity was associated with increased abundance of the Luc protein (Figures 4B and 5C). Auld *et al.* demonstrated that PTC124 increases cellular Luc activity levels by posttranslational stabilization [12]. Thermal stability of the Luc protein was significantly enhanced in the presence of synthetic PTC124-AMP during thermal denaturation assays [12]. However, F-53 was rapidly metabolized and activated to F-53-CoA, probably via the intermediate F-53-AMP, although we did not detect the presence of F-53-AMP itself; therefore, it was surprising to find that F-53-AMP is a stable compound (see the **Production of F-53-CoA by using Luc and microsomes** subsection in the Results section). Therefore, we suggest that lysine-529 regulates enzyme activity as well as controls the translation of Luc transcripts and/or proteolysis of Luc. Some recent studies involving mutants that lack lysine residues known to be targets of acetyltransferases have demonstrated an antagonistic relationship between acetylation and ubiquitination in the regulation of protein stability [33]–[35].

Of particular importance in this study was the demonstration that the ligation of F-53 was induced not *in vitro* but only in living mammalian cells (Figures 4 and 6). Moreover, mouse liver microsomes can generate F-53-CoA *in vitro* (Figure 8). These findings suggest that adduct formation might be induced by any of several mammalian enzymes with ACS activities and might not be restricted exclusively to cells that transiently express Luc (Figure 10). Carboxylic acid-containing drugs, including non-steroidal anti-inflammatory drugs and valproic acid, can be activated by the ACS-catalyzed formation of acyl-CoA thioesters, and have been implicated in rare but serious adverse reactions [22], [23]. Valproic acid, which inhibits rat brain microsomal ACSL4, reduces arachidonic acid turnover; this suggests a possible mechanism that might account for the efficacy of valproic acid in treating bipolar disorder [36]. However, sites of interaction between acyl-CoA and mammalian enzymes have yet to be identified. In this study, however, it is meaningful that the site of ligation of F-53 in living mammalian cells was identified even though the target was the active regulatory lysine of Luc. Lysine residues that resemble lysine-529 of Luc have been identified in the human ACSL protein sequence following alignments obtained using the Basic Local Alignment Search Tool (our unpublished work). We are currently confirming whether human ACSL produces F-53-CoA from F-53. Our future experiments will examine what kind of transferases in mammalian cells transport F-53-CoA. Identification of these transferases will be useful to deal with the aromatic carboxylic acid like F-53 not only in screening assays that use Luc as the reporter, but also in pharmacological and toxicological assays.

In conclusion, we have demonstrated that a new aromatic carboxylic acid abolished Luc enzyme activity in living mammalian cells under the conditions used in the present study. The mechanism involves covalent binding to the enzymatic regulatory lysine in Luc and might be useful for explaining the pharmacological and toxicological effects of the carboxylic acid-containing drugs.
